# A Complex System of Glacial Sub-Refugia Drives Endemic Freshwater Biodiversity on the Tibetan Plateau

**DOI:** 10.1371/journal.pone.0160286

**Published:** 2016-08-08

**Authors:** Catharina Clewing, Christian Albrecht, Thomas Wilke

**Affiliations:** Department of Animal Ecology and Systematics, Justus Liebig University Giessen, Giessen, Germany; National and Kapodistrian University of Athens, GREECE

## Abstract

Although only relatively few freshwater invertebrate families are reported from the Tibetan Plateau, the degree of endemism may be high. Many endemic lineages occur within permafrost areas, raising questions about the existence of isolated intra-plateau glacial refugia. Moreover, if such refugia existed, it might be instructive to learn whether they were associated with lakes or with more dynamic ecosystems such as ponds, wetlands, or springs. To study these hypotheses, we used pulmonate snails of the plateau-wide distributed genus *Radix* as model group and the Lake Donggi Cona drainage system, located in the north-eastern part of the plateau, as model site. First, we performed plateau-wide phylogenetic analyses using mtDNA data to assess the overall relationships of *Radix* populations inhabiting the Lake Donggi Cona system for revealing refugial lineages. We then conducted regional phylogeographical analyses applying a combination of mtDNA and nuclear AFLP markers to infer the local structure and demographic history of the most abundant endemic *Radix* clade for identifying location and type of (sub-)refugia within the drainage system. Our phylogenetic analysis showed a high diversity of *Radix* lineages in the Lake Donggi Cona system. Subsequent phylogeographical analyses of the most abundant endemic clade indicated a habitat-related clustering of genotypes and several Late Pleistocene spatial/demographic expansion events. The most parsimonious explanation for these patterns would be a scenario of an intra-plateau glacial refugium in the Lake Donggi Cona drainage system, which might have consisted of isolated sub-refugia. Though the underlying processes remain unknown, an initial separation of lake and watershed populations could have been triggered by lake-level fluctuations before and during the Last Glacial Maximum. This study inferred the first intra-plateau refugium for freshwater animals on the Tibetan Plateau. It thus sheds new light on the evolutionary history of its endemic taxa and provides important insights into the complex refugial history of a high-altitude ecosystem.

## Introduction

Until recently, the freshwater diversity of the Tibetan Plateau and adjacent areas remained poorly understood. However, a number of phylogenetic and biogeographical studies, conducted in the past years, provided exciting new insights into the evolutionary history of plateau freshwater biota [1], particularly for fishes [2–5], mollusks [6–10], and crustaceans [11,12]. The biogeographical patterns inferred are surprisingly complex: i) only relatively few vertebrate and invertebrate families are present on the plateau, ii) the highest biodiversity can be found in peripheral water bodies, particularly in the major effluent river systems on the southern and eastern plateau, and iii) endemism can be high, depending on the evolutionary and life history of the species involved.

Studies on Tibetan Plateau invertebrates, for example, have shown that taxa with a high passive dispersal capacity, such as amphipods of the genus *Gammarus* [11] and bivalves of the family Sphaeriidae [8], appear to lag pronounced endemism. Moreover, colonization of the plateau likely happened recently, i.e., during the late Pleistocene or Holocene. However, in other taxa, such as the pulmonate snail genera *Gyraulus* [7] and *Radix* [6], the degree of endemism is unexpectedly high. The respective endemic lineages not only occur in peripheral river systems but also under permafrost conditions in relatively isolated intra-plateau areas. Moreover, at least some of these lineages appear to have diverged from their extralimital congeners prior to the Last Glacial Maximum (LGM) [6,7], 25–15 ka BP [13].

This biogeographical pattern–the occurrence of endemic lineages within permafrost areas–parallels patterns in other Palearctic regions that are discussed within the concept of ‘northern glacial refugia’ [14–17]. Accordingly, taxa may have survived the LGM in small, isolated, and temporally ice-free areas within the permafrost zone. Analogous intra-plateau refugial processes are therefore also conceivable for plateau freshwater taxa [6,7]. These potential refugia may include freshwater lakes and other lentic water bodies such as ponds and wetlands, or lotic systems such as (hot) springs. Many plateau lakes are often relatively large, deep and oligotrophic, and might thus have provided suitable conditions for some organisms even if the entire water body or peripheral parts where covered by ice for extended periods of time. In addition, highly dynamic ecosystems (ponds, wetlands, or springs), may have supported seasonably ice-free areas during glacial periods. In the case of hot springs [18,19], such ice-free areas may even have existed throughout the year.

However, it remains unknown whether potential intra-plateau refugia were associated with lakes or peripheral, highly dynamic systems. It is even conceivable that a complex system of sub-refugia, i.e., ‘refugia within refugia’ [20], may have enabled survival of invertebrate populations during glacial periods.

Testing these scenarios is non-trivial as it requires a model study area that fulfills several criteria: i) it should provide suitable freshwater habitats (note that many intra-plateau aquatic systems within endorheic basins are saline or even hyper-saline), ii) it should be limnologically diverse (i.e., contain lakes, ponds, wetlands, springs, small local rivers etc.), and iii) it should be accessible for researchers allowing a reasonable sampling. One of the few freshwater systems on the Tibetan Plateau, satisfying all of these criteria, is the Lake Donggi Cona drainage system [21–23], located in the north-eastern part of the plateau (for more details see the Material and Method section).

In the present study, we examined *Radix* populations from different habitats and locations scattered throughout the Lake Donggi Cona drainage system in order to infer the refugial history of this highly diverse and widespread group of plateau gastropods. Our working hypothesis is that the actual lake has served as a freshwater refugium that allowed both the intralacustrine survival of gastropod populations during glaciation events and the re-colonization of the drainage system after the LGM.

Based on a combination of mitochondrial DNA (mtDNA) data, suitable for addressing older phylogenetic events, as well as genome-wide nuclear DNA (nuDNA) fingerprinting data, reflecting younger phylogeographical and demographic events, we conducted a hierarchical set of analyses.

1. We first performed phylogenetic analyses based on mtDNA data to assess the overall relationships of *Radix* populations inhabiting the Lake Donggi Cona system in a plateau-wide context to identify potential refugial lineages.
2. We then conducted phylogeographical analyses (incl. coalescent approaches) using a combination of mtDNA and nuDNA data to assess the local structure and demographic history of the most abundant endemic *Radix* lineage in the area to infer location and type of (sub-)refugia within the Lake Donggi Cona system.

Our study might not only help biogeographers and evolutionary biologists to better understand the evolution and Pleistocene history of Tibetan Plateau freshwater invertebrates (especially at small spatial scales). It might also provide important insights into the stability and complex refugial structure of isolated high-altitude ecosystems.

## Material and Methods

### Study System and Sampling

**Fig 1:**
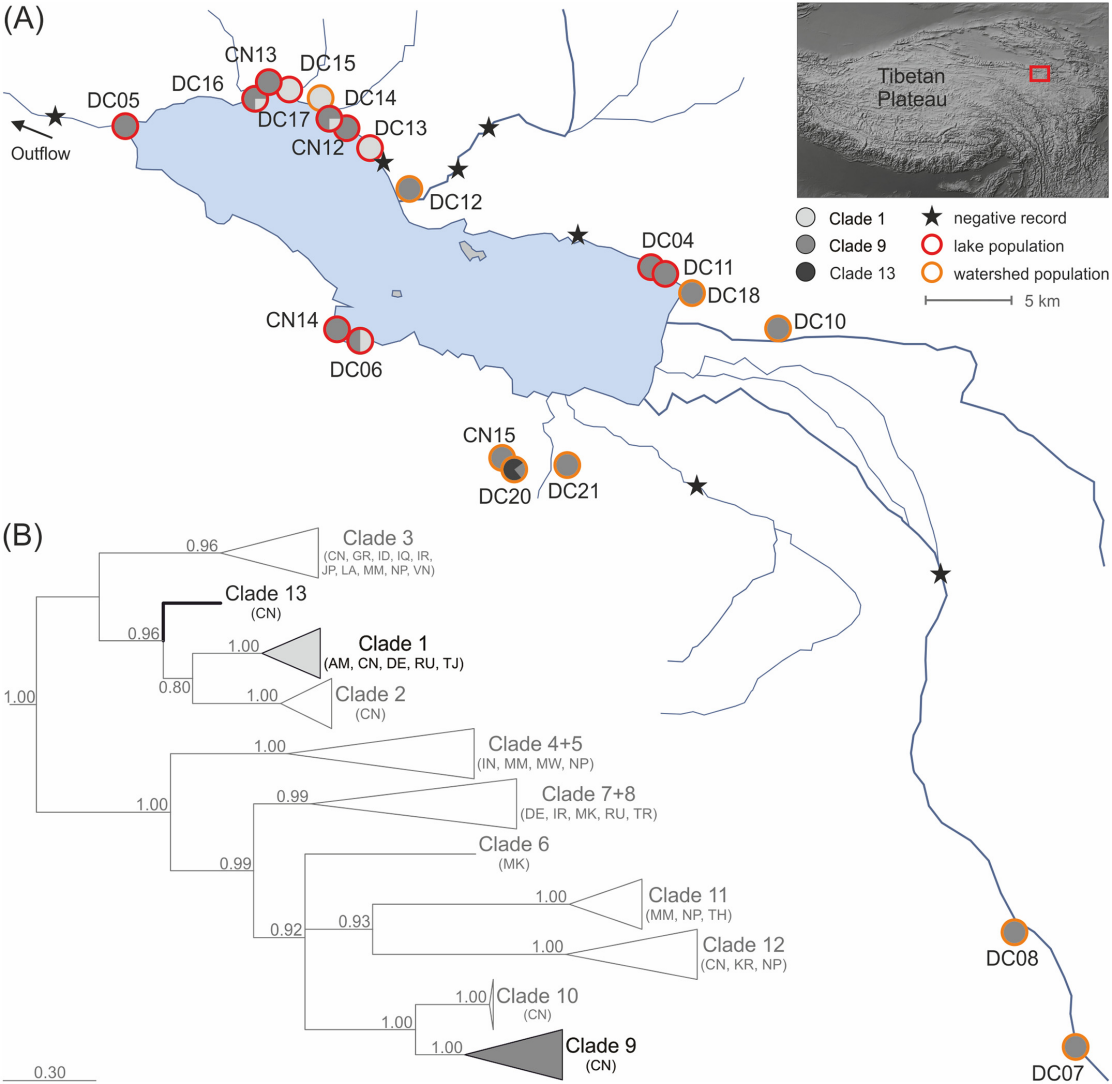
Sampling sites and phylogenetic relationships of *Radix* specimens sampled in the Lake Donggi Cona drainage system. (A) Lake Donggi Cona drainage system showing the lake, the perennial eastern and northeastern inflows (thick lines), some seasonal inflows, and the gauge-controlled outflow. Lake map redrawn from Dietze et al. [21]. Satellite image (upper right corner) taken from Natural Earth (free vector and raster map data at naturalearthdata.com). (B) Collapsed Bayesian-inference tree based on mtDNA (COI) data. The uncollapsed tree is given in the Supplement (see S1 Fig).

The Lake Donggi Cona drainage system (Qinghai Province, People's Republic of China; see Fig 1) is located at an altitude of 4,090 m above sea level (a.s.l.) in the western part of a pull-apart basin [24], belonging to the highly active Kunlun Fault [21]. Oligotrophic Lake Donggi Cona probably emerged during the Pliocene or Early Pleistocene [25,26]. It has a max. depth of 98 m and a surface area of ~229 km^2^ [23]. The lake is fed by perennial inflows in the east and northeast, as well as by seasonal inflows in the north and south. The catchment area has a total size of 3,174 km^2^ [23]. The outflow at the western lake margin is controlled since the 1970s by a gauge station, discharging lake waters towards the endorheic Qaidam Basin ca. 200 km to the northwest [21].

In October 2008 and 2011, specimens of the freshwater gastropod genus *Radix* were collected from a total of 20 sites scattered throughout the Lake Donggi Cona drainage system by handpicking from rocks and water plants or by sieving in shallow waters (see Fig 1). Seven sites did not yield *Radix* snails. Specimens were preserved in 80% ethanol for subsequent analyses. All necessary permits for collecting Tibetan Plateau freshwater mollusks were issued by the Chinese Academy of Sciences (Beijing, China).

### DNA Extraction, Mitochondrial DNA Sequencing, and AFLP Genotyping

Genomic DNA was extracted using the protocol described by Winnepenninckx et al. [27]. For the amplification of the mitochondrial cytochrome *c* oxidase subunit I (COI) gene fragment, the forward primer LCO1490 [28] and the revers primer COR722b [29] were used. Sequences were determined on an ABI 3730 XL sequencer (Life Technologies). The alignment of the protein-coding COI fragment was done by eye, resulting in a final dataset of 655 base pairs (bp) in length. All newly generated sequences are deposited in GenBank (see S1 Table).

AFLP genotyping was performed for a subset of 91 specimens belonging to the most abundant endemic watershed lineage (see Table 1, Fig 1), using a four-step protocol [30,31]:

1. Digestion/ligation reaction; carried out in a total reaction volume of 10 μl containing ddH_2_O, 100x BSA, 10x Ligase buffer, *EcoR*I and *Mse*I adapter (5 μM and 50 μM, respectively), MseI (4 u/μl) and EcoRI (20 u/μl), 400 u/μl T4 ligase, and template DNA (~50 ng).
2. Pre-selective amplification; conducted in a total volume of 13 μl containing ddH_2_O, 10x ThermoPol buffer, dNTPs (2.0 mM each), pre-selective primers (for details see S2 Table; each 75 ng/μl), 2.5 μM MgCl, Taq polymerase (5 u/μl), and ligated products (diluted 1:40).
3. Selective amplification using 14 primer combinations; performed in a total reaction mixture of 12 μl containing ddH_2_O, 10x ThermoPol buffer, dNTPs (2.0 mM each), Taq polymerase (5 u/μl), fluorescent labeled *EcoR*I primers (1 μM each), selective *Mse*I primers (10 μM each; see S2 Table).
4. Gel-electrophoresis; selective amplified fragments were visualized on a capillary sequencer. Fragment scoring was performed with the GeneMarker software v1.90 (Softgenetics), with bands recognized as present (1) or absent (0). Only bands with a length ranging from 100–500 bp and occurring in at least two samples were used to reduce homoplasy and to increase reproducibility [32].

**Table 1:** Locality information for *Radix* populations collected in the Lake Donggi Cona drainage system. It includes locality code (DC = this study; CN = Oheimb et al. [6]), geographical coordinates, clades occurring at the respective locality inferred from the Bayesian-inference analysis (see Fig 1), type of system (L = lake and DS = drainage system), and the number of specimens studied (for populations that contain specimens belonging to different clades, the number of specimens for the most abundant endemic *Radix* clade 9 are shown in brackets).

Locality code	Latitude (in °N)	Longitude (in °E)	Clade no.	System	# specimens studied	
					COI	AFLP
DC04	35.29255	98.69807	9	L	11	12
DC05	35.35419	98.34894	9	L	11	11
DC06	35.24891	98.51102	1, 9	L	10 (5)	-
DC07	34.90175	98.94878	9	DS	10	10
DC08	34.96047	98.91378	9	DS	8	8
DC10	35.25753	98.78893	9	DS	10	10
DC11	35.28861	98.70116	9	L	10	10
DC12	35.33111	98.54857	9	DS	10	10
DC13	35.35374	98.52026	1	L	8	-
DC14	35.37106	98.49651	1, 9	L	8 (6)	-
DC15	35.38115	98.47440	1	L	8	-
DC16	35.38277	98.45560	1, 9	L	4 (3)	-
DC17	35.37305	98.49303	1	DS	6	-
DC18	35.28288	98.71648	9	DS	9	10
DC20	35.19441	98.61356	9, 13	DS	5 (2)	-
DC21	35.19160	98.64289	9	DS	8	10
CN12	35.37019	98.49866	9	L	6	-
CN13	35.38323	98.46711	9	L	6	-
CN14	35.25400	98.50352	9	L	1	-
CN15	35.19938	98.60763	9	DS	7	-
**Total #**					**156 (123)**	**91**

After AFLP genotyping, we tested the reproducibility of our analyses. For this, ~10% of the samples were randomly selected and reanalyzed (steps 1 to 4), using fresh reagents as suggested by Meudt and Clarke [33].

### Phylogenetic Analyses of mtDNA Data

To assess the overall relationships of Lake Donggi Cona *Radix* populations in a plateau-wide context for identifying potential refugial lineages, we complemented our COI data set with data available from GenBank for the Tibetan Plateau, the adjacent Himalaya mountain range, and several locations in Africa and Eurasia. The final dataset comprised 308 *Radix* specimens (120 unique haplotypes) as well as two outgroup taxa: *Planorbarius corneus* (GenBank accession number AY282590) and *Physa fontinalis* (EU818796).

Prior to subsequent analyses, the GTR + I + Γ model was inferred as best-fit substitution model based on the Akaike information criterion using jModelTest v2.1.4 [34]. The COI dataset was tested for substitutional saturation using the test of Xia et al. [35], implemented in DAMBE v5.2.73 [36], with the values for I (proportion of invariant sites) suggested by jModelTest. The test indicated only little saturation: the observed index of substitution saturation (Iss = 0.592) was lower than the critical Iss value (Iss.c = 0.805) under the assumption of a symmetrical topology.

The phylogenetic reconstruction was done in MrBayes v3.2.2 [37] using two parallel runs based on the model inferred by jModelTest as well as the following parameters: nchains = 4, ngen = 5,000,000, samplefreq = 100, temp = 0.1. The combined set of trees from the two runs showed both high effective sample size values (> 920 for all parameters) and a smooth frequency plot as visualized in Tracer v1.5.0 [38].

### Phylogeographical Analyses of mtDNA Data

For subsequent phylogeographical analyses, only specimens were used that belonged to the most abundant endemic Lake Donggi Cona clade (i.e., clade 9, N = 123; see Fig 1), which contained 59 specimens from the actual lake and 64 specimens from the surrounding drainage basin. First, a statistical parsimony network analysis was performed using the software tool TCS v1.21 [39] with a connection limit of 95%.

In order to quantify the degree of genetic structure within and between lake vs. drainage populations, we conducted hierarchical AMOVAs based on 10,000 permutations of the original dataset, using the software package Arlequin v3.5.1.2 [40]. We then assessed the occurrence and timing of significant demographic and spatial expansion events within Lake Donggi Cona specimens as well as within groups of lake and drainage populations using a popular coalescent approach–mismatch distribution analyses–as implemented in Arlequin. The distribution of the observed number of differences between pairs of haplotypes was compared with the simulated distribution under sudden demographic and spatial expansion models. Thereby, unimodal or multimodal distributions of pairwise nucleotide differences indicate expansion events or demographic equilibrium, respectively. The goodness of fit was statistically assessed using the sum of squared differences (SSD; bootstrap replicates = 10,000) between the simulated and observed data. If the SSD *p*-values are below 0.05, the assumption of population expansion (demographic and/or spatial) would have to be rejected [41]. We also calculated Harpending's raggedness index (RI), which is another measure for the goodness of model fit as well as the population parameter τ, which enables the calculation of time since expansion [41]. In the absence of a *Radix*-specific substitution rate for the COI gene, we used the published trait-specific COI Protostomia molecular clock rate (μ) of 0.0122 (95% confidence interval: 0.0095–0.0149) substitutions per site and one million years for the Jukes-Cantor model [42] and the number of nucleotide sites of the COI fragment (n = 655) to estimate the absolute time since expansion in millions of years as t = τ(2μ·n)^-1^ (sensu Rogers and Harpending [43]). To account for the large error of this substitution rate, we calculated t using both the upper and lower bounds of μ.

### Phylogeographical Analyses of AFLP data

A NeighborNet analysis of the AFLP dataset was conducted with the software tool SplitsTree4 v4.13.1 [44]. In order to test for significant structuring between and within groups of lake vs. drainage populations, we performed hierarchical AMOVAs, similar to those conducted for the mtDNA dataset. Additionally, the genetic structure was assessed using a model-based clustering method implemented in the tool STRUCTURE v2.3.4 [45]. The parameters were set as follows: burn-in = 100,000; MCMC iterations = 1,000,000; ancestry model = admixture model; allele frequency model = correlated. We conducted five independent runs with a number of clusters (*K*) ranging from two to twelve. As previously suggested [46], the online-tool structure harvester web v0.6.94 [47] was used to analyze the STRUCTURE results by calculating the rate of change in the probabilities between successive *K* values (Δ*K*). The lowest *K* value with the highest likelihood was then selected and the respective STRUCTURE outputs were averaged with CLUMPP v1.1.1 [48] using a greedy algorithm and pairwise matrix similarity statistics. Results were visualize as bar plots utilizing the software Distruct v1.1 [49].

## Results

### Phylogenetic Analysis of mtDNA Data

The Bayesian inference analysis of 310 specimens revealed that the Lake Donggi Cona drainage system harbors *Radix* specimens belonging to three distinct clades (clades 1, 9, and 13; Fig 1B and S1 Fig). Clade 1 (BPP = 1.0) contained *Radix* specimens from throughout the Palearctic (e.g., Russia, Germany, Tajikistan, Armenia, and China) including the Tibetan Plateau. Within this non-endemic clade, specimens from the Lake Donggi Cona system formed a highly supported sub-clade (BPP = 1.0) together with other plateau specimens. Clade 9 (BPP = 1.0) exclusively contained specimens form the plateau. Within this endemic clade, specimens from the Lake Donggi Cona system build a sub-clade supported by a BPP of 1.0. Finally, endemic clade 13 contained a single haplotype shared by three specimens from the Lake Donggi Cona system (BPP support for the split with the sister group is 0.96).

Lake Donggi Cona snails belonging to the non-endemic clade 1 occurred in 7 out of 20 locations (35%), while the endemic clade 13 was only found in a single location (5%). Most specimens clustered within the endemic clade 9 and originated from a total of 17 locations (85%).

### Phylogeographical Analyses of mtDNA Data

**Fig 2:**
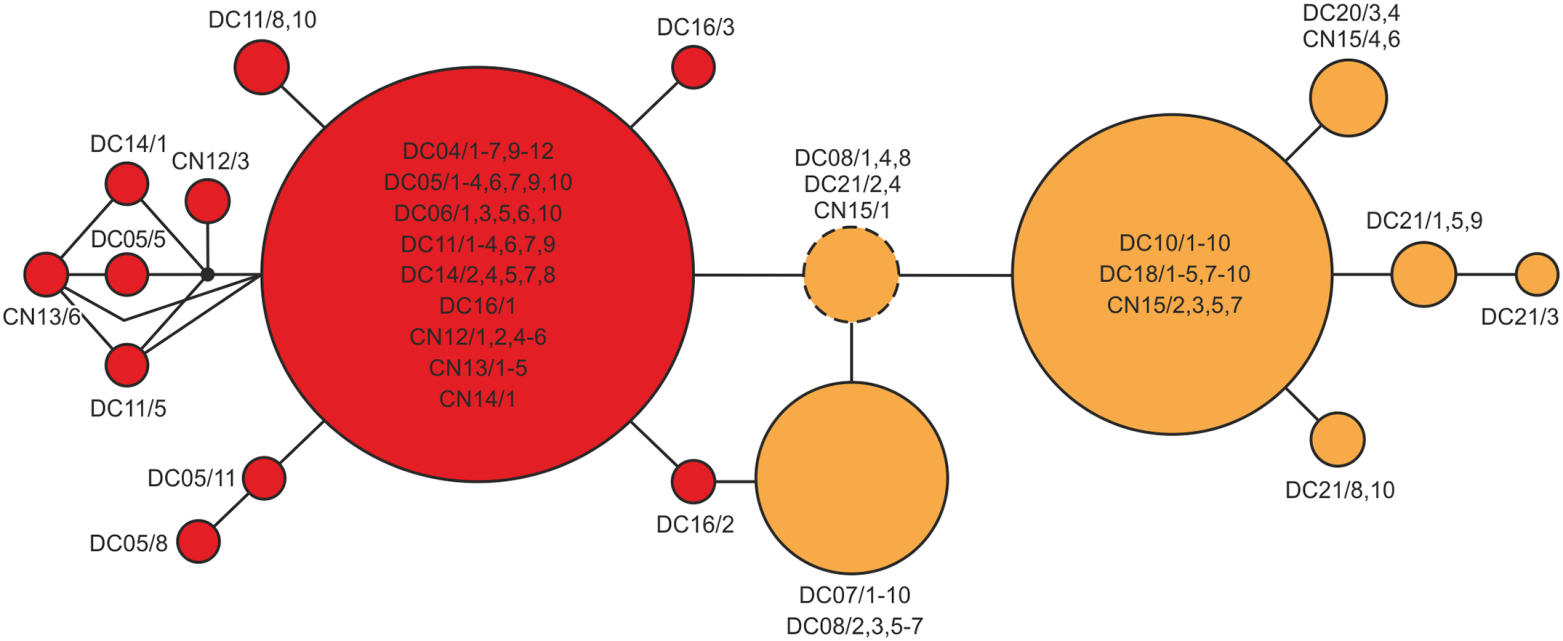
Statistical parsimony network of Lake Donggi Cona *Radix* specimens based on mtDNA data. All haplotypes inferred are endemic to the Lake Donggi Cona drainage system. Red circles: lake specimens, orange circles: drainage specimens. Specimen and location details are given in the Supplement (see S1 Table). The most probable ancestral haplotype is indicated by a dashed line.

The TCS network analysis of the COI dataset for clade 9 (123 specimens) resulted in a single network with 18 haplotypes separated from each other by a maximum of nine mutational steps (see Fig 2). All haplotypes are endemic to the Lake Donggi Cona system. The network revealed two weakly differentiated sub-clusters; no haplotypes were shared between lake (11 haplotypes) and surrounding drainage system populations (7 unique haplotypes). The lake cluster was characterized by a common central haplotype (shared by 42 specimens) to which 10 weakly differentiated haplotypes are connected in a star-like pattern. The drainage-system cluster is characterized by a common central haplotype (shared by 35 specimens) and a common terminal haplotype (shared by 16 haplotypes). These two common haplotypes are linked by the haplotype with the highest probability to be the ancestor and which is shared by five specimens from three drainage locations.

The AMOVA revealed that 64% of the total molecular variation is distributed among and 21% within the two sub-clusters (lake vs. drainage system); about 15% of variation was found within populations (see Table 2).

**Table 2:** Results of the AMOVAs for Lake Donggi Cona *Radix* specimens based on mtDNA and AFLP data. Groups of populations refer to lake vs. drainage populations.

Source of variation	Variation (in %)	*p*-value
**COI data**		
V_a_ (among groups of populations)	64.02	≤ 0.001
V_b_ (within groups of populations)	21.43	≤ 0.001
V_c_ (within populations)	14.56	≤ 0.001
**AFLP data**		
V_a_ (among groups of populations)	7.59	≤ 0.010
V_b_ (within groups of populations)	20.05	≤ 0.001
V_c_ (within populations)	72.34	≤ 0.001

The mismatch analysis of all specimens from the Lake Donggi Cona drainage system resulted in SSD *p*-values below 0.05 (Table 3). Therefore, the assumption of demographic and/or spatial population expansions for the entire system had to be rejected [41]. For the group of lake specimens, a sudden demographic expansion had to be rejected as well due to significant SSD *p*-values. However, the spatial expansion assumption was not rejected. This is also true for both sudden demographic and spatial expansion assumptions in the group of drainage specimens. When comparing the absolute values of τ for the spatial extension events in groups of lake vs. drainage specimens, then the value was higher for the latter one (Table 3), indicating that the spatial extension of drainage specimens started prior to the spatial extension of lake specimens. Preliminary calculations of the absolute timing of these three significant expansion events showed that the spatial expansion of the lake populations started between 48,000 and 30,100 years ago (lower and upper bound of μ, respectively); the spatial expansion of the drainage populations between 121,100 and 77,200 years ago, and the demographic expansion of the drainage populations between 120,000 and 76,500 years ago. Note that these values only account for the error of μ and not for the CI of τ (see Table 3).

**Table 3:** Results of mismatch distribution analyses for Lake Donggi Cona *Radix* specimens based on mtDNA. SSD = sum of squared deviations from the respective model; RI = raggedness index; τ = population parameter tau with 95% confidence interval; *P* = significance of the respective parameter (level 0.05).

	Spatial expansion						Sudden demographic expansion					
	SSD	*P* (SSD)	RI	*P* (RI)	τ	τ (CI)	SSD	*P* (SSD)	RI	*P* (RI)	τ	τ (CI)
**“All”**	0.0429	**(0.024)**	0.1616	(0.056)	1.994	(0.444–3.492)	0.0476	**(0.033)**	0.1616	(0.007)	1.990	(0.621–3.439)
**Lake**	0.0002	(0.740)	0.2360	(0.597)	0.597	(0.000–2.924)	0.0428	**(0.046)**	0.5422	(0.603)	3.000	(0.387–3.500)
**Drainage**	0.0096	(0.357)	0.0639	(0.699)	1.507	(0.300–3.129)	0.0071	(0.382)	0.0532	(0.751)	1.494	(0.000–3.262)

### Phylogeographical Analyses of AFLP data

**Fig 3:**
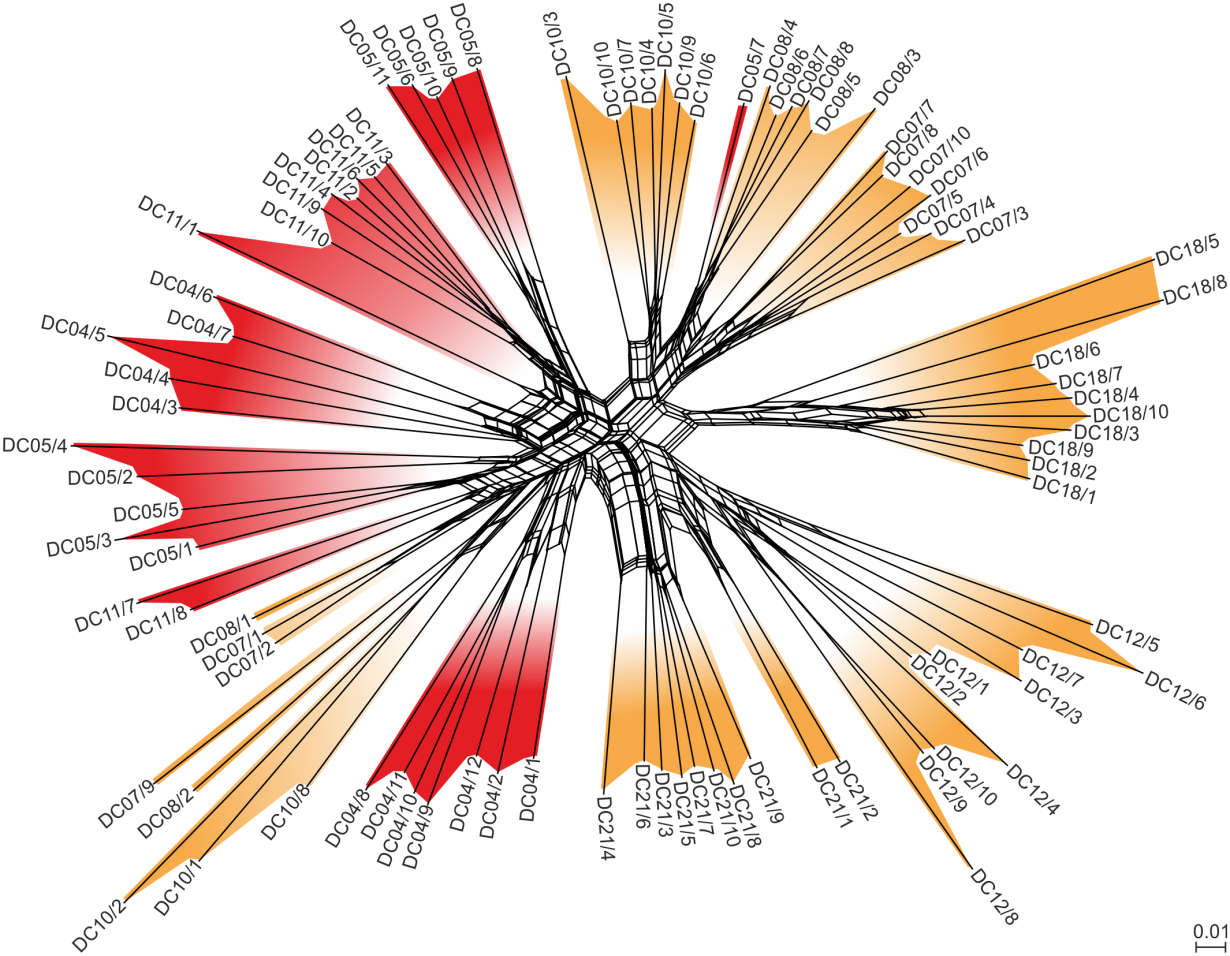
NeighborNet of Lake Donggi Cona *Radix* specimens based on AFLP data. Lake specimens are colored in red; drainage specimens in orange. For specimen and locality details see the Supplement (S1 Table).

The average reproducibility of the AFLP data in the present study was 77.5% (ranging from 70.8% to 84.4%). The AFLP NeighborNet analysis of 91 specimens revealed some degree of structuring of lake vs. drainage system populations (see Fig 3). However, a total of 8 specimens of the drainage system populations (DC10/1–2, 8, DC08/1–2, DC07/1–2, 9) clustered within the lake group, while lake-specimen DC05/7 clustered within the drainage group.

**Fig 4:**
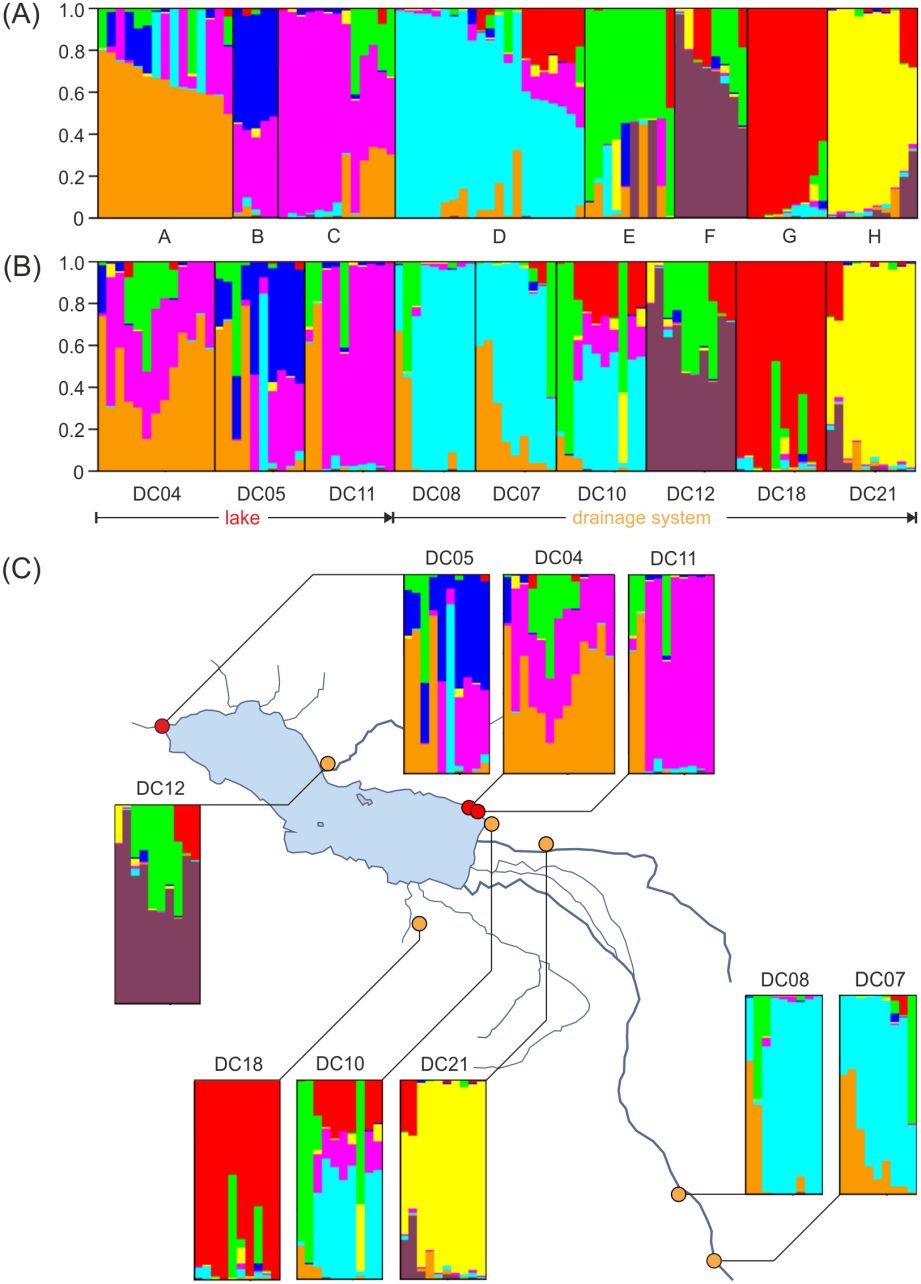
STRUCTURE output (K = 8) for Lake Donggi Cona *Radix* specimens based on AFLP data. Each group assignment is represented by a colored horizontal bar. (A) Accessions ordered according to their cluster membership (K = 8: A–H). (B) Accessions ordered according to their sampling site (lake: DC04, 05, 11; drainage system: DC07, 08, 10, 12, 18, 21). (C) Geographical presentation of the structure results. Red circles: sampling sites within the lake, orange circles: sampling sites within the drainage system. Lake map redrawn from Dietze et al. [21].

The AMOVA showed that 8% of the total molecular variation was distributed among groups (lake vs. drainage system), 20% within groups, and 72% within populations (see Table 2). The software STRUCTURE suggested eight as the most probable number of clusters for the populations studied, indicating substantial structuring (see Fig 4A). Nonetheless, the three lake populations were relatively similar and clustered together (Fig 4B and 4C). In contrast, the drainage populations were more divers with some but not all populations clustering together.

## Discussion

In this study we examined *Radix* populations from the Lake Donggi Cona drainage system in order to unravel the refugial history of plateau freshwater organisms. Our working hypothesis was that the actual lake served as a freshwater refugium, supporting the survival of gastropod populations during glacial phases and enabling the re-colonization of the drainage system after the LGM.

Specifically, we conducted phylogenetic analyses based on mtDNA data to assess the relationships of *Radix* populations from the Lake Donggi Cona system with other plateau and non-plateau populations for identifying potential refugial lineages. Then, we performed phylogeographical analyses based on mtDNA and nuDNA data, using specimens of the most common refugial clade (i.e., clade 9) to assess their population structure and demographic history. These data, in turn, might be informative for inferring location and type of potential sub-refugia within the drainage system.

### Identification of potential refugial *Radix* lineages inhabiting the Lake Donggi Cona System

Our phylogenetic analysis of 310 specimens indicated that the diversity of *Radix* spp. in the Lake Donggi Cona system is surprisingly high and that the respective specimens belong to three well-supported, unrelated clades (clades 1, 9, 13). Moreover, the degrees of plateau endemicity and even narrow-range endemicity (i.e., genetic lineages endemic to the Lake Donggi Cona system) are significant. Within clade 1, Lake Donggi Cona specimens form a highly supported endemic sub-clade together with other plateau specimens (see S1 Fig). Within endemic clade 9, containing most of the Lake Donggi Cona specimens, the local individuals form a highly supported (BPP = 1.0) sub-clade of narrow-range endemics. Moreover, all clade-13 specimens appear to be narrow-range endemics.

These patterns of endemicity are not in concordance with those previously observed for Palearctic *Radix* populations occurring outside the plateau [50]. Freshwater mollusks such as *Radix* spp. are known for their high passive dispersal capacity, particularly along river systems [51] and bird migration routes [50]. Post-glacial recolonization patterns are therefore typically associated with i) a strong admixture of genotypes across a species’ range (i.e., lack of isolation-by-distance) and ii) a genetic impoverishment of local populations (due to genetic drift) [52]. The latter process is likely reinforced by the ephemeral character of many *Radix* habitats [52,53] and potentially also by increased selfing of mixed-mating animals in newly colonized areas ([52]; but see also Bousset et al. [54]).

Therefore, the high divergence and phylogenetic distinctness of local *Radix* clades, on the one hand, and the considerable genetic diversity within populations, on the other hand, found in the current study are not consistent with a scenario of post-glacial recolonization of the Lake Donggi Cona drainage system. Although these patterns, in theory, could also have resulted from multiple colonization events from different areas and/or during different times [55–57], this would not explain the star-like patterns observed in the haplotype network (see Fig 2) and the distinct habitat-related structuring of local *Radix* populations indicative of local adaptation (see Table 2, Figs 2 and 3). The most parsimonious explanation for the patterns observed is therefore a scenario of an intra-plateau refugium in the Lake Donggi Cona drainage system that enabled the survival of freshwater mollusks on the plateau in a highly isolated watershed (sensu Hewitt [58]). The pattern of several endemic clades and sub-clades occurring in the area also suggests that the refugial history of this lake system might have been very complex, probably involving a pre-LGM colonization by *Radix* specimens originating from different areas outside the plateau (for more information on the timing of demographic events see the section “Location and type of (sub-)refugia within the Lake Donggi Cona system” below).

Unfortunately, it remains challenging to discuss these findings in a comparative context due to the low number of plateau-wide distributed freshwater species and the virtual absence of respective phylogeographical data. Therefore, our current understanding of the refugial history of the Tibetan Plateau is largely based on data from terrestrial species [1,59]. Most previous studies on terrestrial plants indicated the existence of Pleistocene refugia at the southern and southeastern edge of the plateau (reviewed in Zhou et al. [60]). Only recently, the existence of old Pleistocene refugia in the central parts of the plateau was confirmed for a flowering shrub species [61]. In contrast, the majority of studies on terrestrial animals suggested the existence of eastern or northeastern Pleistocene refugia in lower-altitude areas off the platform margins [62,63]. Similar inferences were also made for plateau freshwater vertebrates. Accordingly, some fish species that are today widespread on the plateau [64] as well as the endemic brown frog *Rana kukunoris* [60] may have survived the Pleistocene in riverine refugia beyond the northeastern edge of the plateau.

Comparing these findings with the results of the current study, we suggest that the Lake Donggi Cona drainage system might be the first intra-plateau refugium inferred for freshwater animals, in general, and for invertebrates, in particular. Moreover, the survival of the LGM in a region where glaciers might have descended to altitudes as low as 3,500 m a.s.l. [65] indicates that there were local ice-free areas at least during the summer months. In addition, the habitat-related clustering of genotypes suggests the existence of distinct sub-refugia within the Lake Donggi Cona drainage system.

### Location and type of (sub-)refugia within the Lake Donggi Cona system

Phylogeographical analyses were performed for specimens of the most abundant endemic Lake Donggi Cona *Radix* clade in order to test for location and type of sub-refugia (lake vs. drainage). Our working hypothesis was that the actual lake served as a freshwater refugium. Accordingly, we would have expected to observe i) a higher genetic diversity among lake populations, ii) no clear structuring of lake vs. drainage populations, and iii) possibly derived haplotypes in the drainage system.

However, the phylogeographical patterns inferred are not consistent with these operational criteria. The mtDNA data resulted in a network in which lake and drainage populations form distinct sub-clusters. Though the number of mutational steps between the sub-clusters is low, there are no shared haplotypes among them (Fig 2). This separation of lake and drainage populations is also supported by the AMOVA, which showed the highest partitioning of molecular variation (64%) between lake and drainage clusters (Table 2). The mismatch distribution analyses indicated significant sudden spatial expansion events in both groups during the Late Pleistocene. However, the assumption of a sudden demographic expansion had to be rejected for the lake populations. Moreover, a comparison of τ values (Table 3) even suggested that the spatial extension event of drainage specimens predated that of lake specimens.

These patterns of distinct phylogeographical histories of lake vs. drainage populations are, in principle, confirmed by our nuDNA data, although the AFLP-based structures appear to be less pronounced. In the NeighborNet (Fig 3), some drainage populations clustered within the group of lake populations. Moreover, the variance between lake and drainage populations in the AMOVA (Table 2), although significant, was relatively low with 8%. Finally, the STRUCTURE analyses did not suggest two (lake vs. drainage populations) but eight genetic clusters, though the lake populations were more similar to each other than to the drainage populations (see Fig 4C).

However, the differences between the patterns inferred from the mtDNA and nuDNA datasets are not surprising as the highly variable AFLP data (which target variable non-coding regions with a high substitution rate) typically reflect more recent evolutionary processes compared to mtDNA data [66]. One part of the problem might also be the comparably low reproducibility of our AFLP analyses. Unfortunately, AFLP reproducibility problems are common when using materials that could not have been preserved under ideal conditions, thus affecting DNA quality [67,68].

Although the mtDNA and nuDNA pattern are not fully in concordance, both suggest a relative distinctness of lake vs. drainage populations. Therefore, our working hypothesis that the actual lake served as (the sole) refugium has to be rejected. Instead, the patterns inferred might be better explained by a scenario in which the lake and the surrounding drainage system provided isolated sub-refugia (sensu Shafer et al. [20]) for *Radix* populations during the last glaciations. Though the underlying processes remain unknown, an initial Pleistocene separation of lake and watershed populations could have been triggered by strong lake-level fluctuations (i.e., up to 39 m below present lake level) before and during the LGM, likely caused by a desiccation of major Lake Donggi Cona inflows [21]. These environmental changes may not only have caused vicariance event(s), but potentially also bottle necks in either group of populations. After environmental recovery, populations may have re-started to diversify. These assumed processes are consistent with the somewhat star-like structure of the two sub-clusters in the mtDNA network (Fig 2) and the results of the mismatch analyses.

On first sight, the AFLP data (which likely reflect more recent phylogeographical and demographic events compared to mtDNA) may suggest a ‘leveling-out’ of an initial vicariance-driven structure of lake vs. drainage populations as seen in the mtDNA data. These changes over time could have been caused by, for example, increasing gene flow (see the relatively low among-group variation in the AMOVA of the AFLP data). However, a closer look at the NeighborNet, and particularly at the STRUCTURE outputs (Figs 3 and 4), might suggest a more complex picture provided by the AFLP data. Accordingly, the lake populations are relatively homogeneous (see populations DC04, DC05, and DC11 in Fig 4C). However, there are considerable differences among drainage populations (see the southeastern populations DC08 and DC07 vs. the northern population DC12 vs. the central populations DC18, DC10, DC21 in Fig 4C). This surprisingly high degree of fine-scale structuring indicates that isolation processes in *Radix* populations from the Lake Donggi Cona drainage system may have continued after the LGM. It also suggests that an initial system of Pleistocene sub-refugia has resulted in complex patterns of biodiversity potentially driven by adaptation and isolation processes.

### Limitations and Outlook

Our study combined nuDNA and mtDNA data from a relatively large number of specimens of Tibetan *Radix* and thus belongs to the most comprehensive analyses of plateau-wide distributed animals. Nonetheless, we have to note three limitations of our approach. First, the reproducibility of the AFLP data is relatively low (see above). This might be related to the sub-optimal sampling and preservation conditions during expeditions to the plateau. In addition, our highly conserved test for reproducibility (i.e., a re-analysis of ca. 10% of the samples with fresh reagents [33]) may have introduced an artificial error due to the different batch of reactions used for the re-analysis [69]. Overall, we believe that the reproducibility problem did not significantly affect the outcome of our study. First, mtDNA and nuDNA are largely concordant. Also, a strong reproducibility problem would have introduced considerable noise into our AFLP dataset. However, the overall structure of the AFLP data is considerable and we even see a remarkable fine-scale structuring among groups of drainage populations (see Fig 4C).

The second problem relates to the fact that the quality of refugial reconstructions largely depends on a comprehensive sampling design outside the respective refugium. The Lake Donggi Cona area is located in the northeastern part of the Tibetan Plateau. Though *Radix* spp. belong to the best-studied freshwater gastropods worldwide, little is known about their occurrences north and northeast of the plateau (see, for example, the large gap for *Radix* records in GBIF [70]). In our study, we were able to include 17 populations from circum-plateau areas. This is not enough to cover the entire regional *Radix* biodiversity and there is, in principle, a chance that some of the endemic haplotypes found in the Lake Donggi Cona area may also occur outside. However, the distinct patterns found in the mtDNA network (see Fig 2) do not support such a scenario. We see private haplotypes in drainage vs. lake populations with one central haplotype each. Such a pattern would be very difficult to explain with a recent introduction of specimens from peripheral areas and the most parsimonious interpretation is an *in-situ* evolution of genetic diversity.

The third problem concerns the lack of calibration events for molecular clock inferences as well as of *Radix*-specific substitution rates for the mismatch analyses. As selfing frequently occurs in *Radix*, it is not yet clear whether commonly accepted substitution rates for (dioeceous) animals [42,71] work in this taxon [72]. Moreover, the high intensity of cosmic radiation on the plateau may affect substitution rates in indigenous taxa [73–75]. In contrast to, for example, UV radiation, this high-energy cosmic radiation penetrates deep into water [76] and may thus affect freshwater animals. However, the lengths of the terminal branches of plateau *Radix* specimens in our phylogenetic analysis (see Supplement; S1 Fig) do not appear to be extended compared to non-plateau specimens. Therefore, a significant cosmic-radiation driven increase in substitution rates in plateau taxa appears to be unlikely. Nonetheless, in the interests of precision and to avoid problems of interpretation, we refrain from linking the estimated absolute timing of demographic events in Lake Donggi Cona *Radix* to discrete glaciation cycles. Although this might not have affected the major inferences of our paper, we do encourage further studies on DNA substitution rates in plateau animals, which may subsequently help providing a better temporal resolution of evolutionary events.

As we were able to infer the first intra-plateau refugium for freshwater animals, we also suggest future studies of other plateau animals and/or other isolated drainage systems. Such studies might help to generate a more comprehensive picture about the consequences of Pleistocene glaciation, in general, and the role of intra-plateau (sub-)refugia, in particular, for generating the exiting patterns of endemic biodiversity seen on the ‘Roof of the World’.

### Conclusions

Our comparative phylogenetic analysis showed that the Lake Donggi Cona system is inhabited by *Radix* individuals belonging to three well-supported, unrelated clades (clades 1, 9, 13). These clades either consist exclusively of Lake Donggi Cona narrow-range endemics or Lake Donggi Cona specimens form sub-clades of endemics/narrow-range endemics. The most parsimonious explanation for these patterns involves a scenario of an intra-plateau refugium in the Lake Donggi Cona drainage system. This assumption is confirmed by detailed phylogeographical and demographic studies of *Radix* specimens that belong to the most abundant endemic *Radix* clade. Within this clade, the mtDNA network analysis indicates distinct clusters of lake vs. drainage populations. These findings are also supported by the AMOVA and by the results of the mismatch distribution analyses, which suggested distinct spatial extension events in both group during the Late Pleistocene. These results are not consistent with the operational criteria for our working hypothesis. Therefore, the hypothesis assuming that Lake Donggi Cona served as freshwater refugium, has to be rejected. Instead, a complex system of sub-refugia, i.e., ‘refugia within refugia’, both within the lake and the surrounding watershed is assumed.

These findings are largely confirmed by our AFLP data, although the latter show a more complex picture. In the NeighborNet, some drainage populations clustered within the group of lake populations and the variance between lake and drainage population in the AMOVA, although significant, was relatively low. Moreover, the STRUCTURE analysis did not suggest two (lake vs. drainage populations) but eight genetic clusters with a considerable degree of fine-scale structure, particularly among drainage populations. A possible explanation would be that a Pleistocene vicariance event, potentially driven by strong lake-level fluctuations, caused an initial separation of lake and watershed populations. However, isolation processes in *Radix* populations form the Lake Donggi Cona drainage system may have continued after the LGM, resulting in the complex patterns of biodiversity that can be observed today.

This study inferred the first intra-plateau refugium for freshwater animals on the Tibetan Plateau and thus sheds new light on the evolutionary history of its endemic taxa. We are confident that future research will likely unravel glacial refugia for other plateau freshwater animals and in other isolated intra-plateau areas. This will subsequently help to obtain a more comprehensive picture about evolutionary processes on the ‘Roof of the World’.

## Supporting Information

S1 Fig Bayesian-inference tree of *Radix* specimens based on the mitochondrial COI gene. Specimens inhabiting the Lake Donggi Cona drainage system are highlighted in bold and are labeled with haplotype codes (for details see S1 Table); remaining sequences with GenBank accession numbers. Major clades are labeled with bars according to the phylogeny of Oheimb et al. [6]. Black bars indicate clades that contain *Radix* spp. from the Lake Donggi Cona drainage system; an asterisk indicates Tibetan Plateau endemic clades. Bayesian posterior probabilities (BPP) are given next to the respective node when BPP were higher than 0.5. The scale bar represents substitutions per side according to the applied model of sequence evolution.
(TIF)

S1 Table List of *Radix* specimens studied. It includes haplotype number, system (L = lake, DS = drainage system), geographical coordinates, haplotype code, clade number according to the phylogenetic analysis, DNA voucher number, and GenBank accession numbers. Specimens used for the AFLP analyses are marked with √.
(DOCX)

S2 Table Information on adapters and PCR primer sequences used for AFLP genotyping *Radix* specimens. (DOCX)
