# Supplementary figures and images for: A Complex System of Glacial Sub-Refugia Drives Endemic Freshwater Biodiversity on the Tibetan Plateau
Source: PLoS One. 2016 Aug 8;11(8):e0160286. doi: 10.1371/journal.pone.0160286 (PMC4976922; doi:10.1371/journal.pone.0160286)

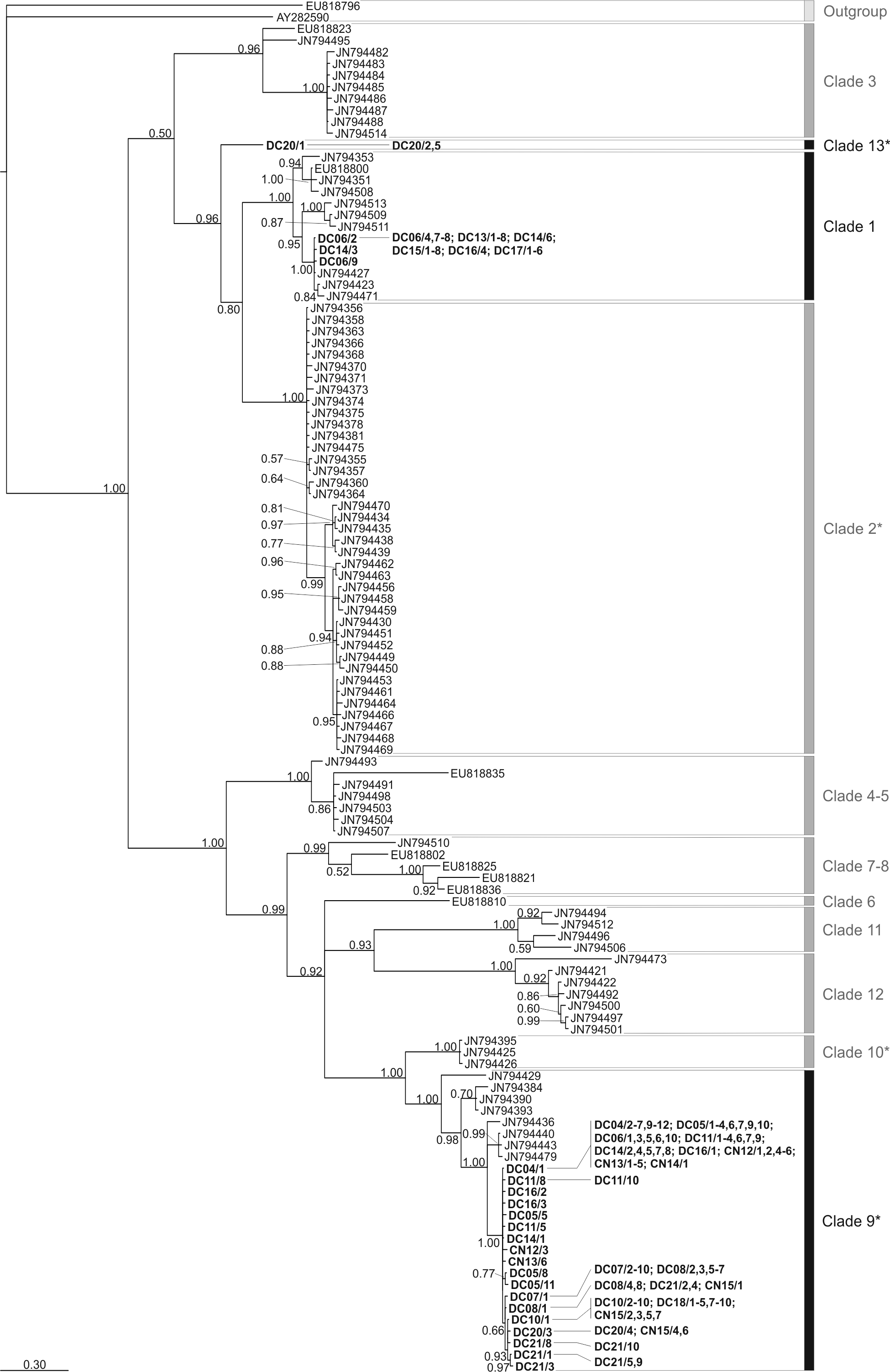

Supplement: S1 Fig — Specimens inhabiting the Lake Donggi Cona drainage system are highlighted in bold and are labeled with haplotype codes (for details see S1 Table); remaining sequences with GenBank accession numbers. Major clades are labeled with bars according to the phylogeny of Oheimb et al. [6]. Black bars indicate clades that contain Radix spp. from the Lake Donggi Cona drainage system; an asterisk indicates Tibetan Plateau endemic clades. Bayesian posterior probabilities (BPP) are given next to the respective node when BPP were higher than 0.5. The scale bar represents substitutions per side according to the applied model of sequence evolution. (TIF) [file pone.0160286.s001.tif]
