# Supplementary material for: A Complex System of Glacial Sub-Refugia Drives Endemic Freshwater Biodiversity on the Tibetan Plateau
Source: PLoS One. 2016 Aug 8;11(8):e0160286. doi: 10.1371/journal.pone.0160286 (PMC4976922; doi:10.1371/journal.pone.0160286)
Supplement: S1 Table — It includes haplotype number, system (L = lake, DS = drainage system), geographical coordinates, haplotype code, clade number according to the phylogenetic analysis, DNA voucher number, and GenBank accession numbers. Specimens used for the AFLP analyses are marked with √. (DOCX) [file pone.0160286.s002.docx]

| Location code | System | Coordinates (°N / °E) | Haplotype code | Clade no. | DNA voucher | UGSB no. | AFLPs | COI GenBank accession no. |
| --- | --- | --- | --- | --- | --- | --- | --- | --- |
| DC04 | L | 35.29255 / 98.69807 | DC04/1 | 9 | 17559 | UGSB 9289 | √ | KX585069 |
|  |  |  | DC04/2 | 9 | 17560 | UGSB 9290 | √ | KX585073 |
|  |  |  | DC04/3 | 9 | 17561 | UGSB 9291 | √ | KX585074 |
|  |  |  | DC04/4 | 9 | 17562 | UGSB 9292 | √ | KX585075 |
|  |  |  | DC04/5 | 9 | 17563 | UGSB 9293 | √ | KX585076 |
|  |  |  | DC04/6 | 9 | 17564 | UGSB 9294 | √ | KX585077 |
|  |  |  | DC04/7 | 9 | 17565 | UGSB 9295 | √ | KX585078 |
|  |  |  | DC04/8 | 9 | 17566 | UGSB 9296 | √ | - |
|  |  |  | DC04/9 | 9 | 18158 | UGSB 10152 | √ | KX585079 |
|  |  |  | DC04/10 | 9 | 18159 | UGSB 10153 | √ | KX585070 |
|  |  |  | DC04/11 | 9 | 18160 | UGSB 10154 | √ | KX585071 |
|  |  |  | DC04/12 | 9 | 18161 | UGSB 10155 | √ | KX585072 |
| DC05 | L | 35.35419 / 98.34894 | DC05/1 | 9 | 17371 | UGSB 8969 | √ | KX585080 |
|  |  |  | DC05/2 | 9 | 17372 | UGSB 8970 | √ | KX585083 |
|  |  |  | DC05/3 | 9 | 17373 | UGSB 8971 | √ | KX585084 |
|  |  |  | DC05/4 | 9 | 17374 | UGSB 8972 | √ | KX585085 |
|  |  |  | DC05/5 | 9 | 17490 | UGSB 9102 | √ | KX585086 |
|  |  |  | DC05/6 | 9 | 18164 | UGSB 10158 | √ | KX585087 |
|  |  |  | DC05/7 | 9 | 18166 | UGSB 10160 | √ | KX585088 |
|  |  |  | DC05/8 | 9 | 18167 | UGSB 10161 | √ | KX585089 |
|  |  |  | DC05/9 | 9 | 18168 | UGSB 10162 | √ | KX585090 |
|  |  |  | DC05/10 | 9 | 18169 | UGSB 10163 | √ | KX585081 |
|  |  |  | DC05/11 | 9 | 18750 | UGSB 11034 | √ | KX585082 |
| DC06 | L | 35.24891 / 98.51102 | DC06/1 | 9 | 17375 | UGSB 8973 |  | KX585091 |
|  |  |  | DC06/2 | 1 | 17376 | UGSB 8974 |  | KX585093 |
|  |  |  | DC06/3 | 9 | 17377 | UGSB 8975 |  | KX585094 |
|  |  |  | DC06/4 | 1 | 17378 | UGSB 8976 |  | KX585095 |
|  |  |  | DC06/5 | 9 | 17601 | UGSB 9330 |  | KX585096 |
|  |  |  | DC06/6 | 9 | 17602 | UGSB 9332 |  | KX585097 |
|  |  |  | DC06/7 | 1 | 17603 | UGSB 9333 |  | KX585098 |
|  |  |  | DC06/8 | 1 | 17604 | UGSB 9334 |  | KX585099 |
|  |  |  | DC06/9 | 1 | 17605 | UGSB 9335 |  | KX585100 |
|  |  |  | DC06/10 | 9 | 17606 | UGSB 9336 |  | KX585092 |
| DC07 | DS | 34.90175 / 98.94878 | DC07/1 | 9 | 17379 | UGSB 8977 | √ | KX585101 |
|  |  |  | DC07/2 | 9 | 17380 | UGSB 8978 | √ | KX585103 |
|  |  |  | DC07/3 | 9 | 17381 | UGSB 8979 | √ | KX585104 |
|  |  |  | DC07/4 | 9 | 17382 | UGSB 8980 | √ | KX585105 |
|  |  |  | DC07/5 | 9 | 18176 | UGSB 10170 | √ | KX585106 |
|  |  |  | DC07/6 | 9 | 18178 | UGSB 10172 | √ | KX585107 |
|  |  |  | DC07/7 | 9 | 18179 | UGSB 10173 | √ | KX585108 |
|  |  |  | DC07/8 | 9 | 18180 | UGSB 10174 | √ | KX585109 |
|  |  |  | DC07/9 | 9 | 18181 | UGSB 10175 | √ | KX585110 |
|  |  |  | DC07/10 | 9 | 18752 | UGSB 11036 | √ | KX585102 |
| DC08 | DS | 34.96047 / 98.91378 | DC08/1 | 9 | 17383 | UGSB 8981 | √ | KX585111 |
|  |  |  | DC08/2 | 9 | 17384 | UGSB 8982 | √ | KX585112 |
|  |  |  | DC08/3 | 9 | 17385 | UGSB 8983 | √ | KX585113 |
|  |  |  | DC08/4 | 9 | 17386 | UGSB 8984 | √ | KX585114 |
|  |  |  | DC08/5 | 9 | 17508 | UGSB 9120 | √ | KX585115 |
|  |  |  | DC08/6 | 9 | 18186 | UGSB 10180 | √ | KX585116 |
|  |  |  | DC08/7 | 9 | 18773 | UGSB 11057 | √ | KX585117 |
|  |  |  | DC08/8 | 9 | 18774 | UGSB 11058 | √ | KX585118 |
| DC10 | DS | 35.25753 / 98.78893 | DC10/1 | 9 | 17387 | UGSB 8985 | √ | KX585119 |
|  |  |  | DC10/2 | 9 | 17388 | UGSB 8986 | √ | KX585121 |
|  |  |  | DC10/3 | 9 | 17389 | UGSB 8987 | √ | KX585122 |
|  |  |  | DC10/4 | 9 | 17390 | UGSB 8988 | √ | KX585123 |
|  |  |  | DC10/5 | 9 | 17607 | UGSB 9337 | √ | KX585124 |
|  |  |  | DC10/6 | 9 | 17608 | UGSB 9338 | √ | KX585125 |
|  |  |  | DC10/7 | 9 | 17609 | UGSB 9339 | √ | KX585126 |
|  |  |  | DC10/8 | 9 | 17610 | UGSB 9340 | √ | KX585127 |
|  |  |  | DC10/9 | 9 | 18188 | UGSB 10182 | √ | KX585128 |
|  |  |  | DC10/10 | 9 | 18189 | UGSB 10183 | √ | KX585120 |
| DC11 | L | 35.28861 / 98.70116 | DC11/1 | 9 | 17611 | UGSB 9341 | √ | KX585130 |
|  |  |  | DC11/2 | 9 | 17612 | UGSB 9341 | √ | KX585131 |
|  |  |  | DC11/3 | 9 | 17613 | UGSB 9342 | √ | KX585132 |
|  |  |  | DC11/4 | 9 | 17614 | UGSB 9343 | √ | KX585133 |
|  |  |  | DC11/5 | 9 | 18194 | UGSB 10188 | √ | KX585134 |
|  |  |  | DC11/6 | 9 | 18195 | UGSB 10189 | √ | KX585135 |
|  |  |  | DC11/7 | 9 | 17391 | UGSB 8989 | √ | KX585136 |
|  |  |  | DC11/8 | 9 | 17392 | UGSB 8990 | √ | KX585137 |
|  |  |  | DC11/9 | 9 | 17393 | UGSB 8991 | √ | KX585138 |
|  |  |  | DC11/10 | 9 | 17394 | UGSB 8992 | √ | KX585129 |
| DC12 | DS | 35.33111 / 98.54857 | DC12/1 | 9 | 17395 | UGSB 8993 | √ | KX585139 |
|  |  |  | DC12/2 | 9 | 17396 | UGSB 8994 | √ | KX585141 |
|  |  |  | DC12/3 | 9 | 17397 | UGSB 8995 | √ | KX585142 |
|  |  |  | DC12/4 | 9 | 17398 | UGSB 8996 | √ | KX585143 |
|  |  |  | DC12/5 | 9 | 17615 | UGSB 9345 | √ | KX585144 |
|  |  |  | DC12/6 | 9 | 17616 | UGSB 9346 | √ | KX585145 |
|  |  |  | DC12/7 | 9 | 17617 | UGSB 9347 | √ | KX585146 |
|  |  |  | DC12/8 | 9 | 17618 | UGSB 9348 | √ | KX585147 |
|  |  |  | DC12/9 | 9 | 18758 | UGSB 11042 | √ | KX585148 |
|  |  |  | DC12/10 | 9 | 18759 | UGSB 11043 | √ | KX585140 |
| DC13 | L | 35.35374 / 98.52026 | DC13/1 | 1 | 17567 | UGSB 9297 |  | KX585149 |
|  |  |  | DC13/2 | 1 | 17568 | UGSB 9298 |  | KX585150 |
|  |  |  | DC13/3 | 1 | 17569 | UGSB 9299 |  | KX585151 |
|  |  |  | DC13/4 | 1 | 17570 | UGSB 9300 |  | KX585152 |
|  |  |  | DC13/5 | 1 | 17571 | UGSB 9301 |  | KX585153 |
|  |  |  | DC13/6 | 1 | 17572 | UGSB 9302 |  | KX585154 |
|  |  |  | DC13/7 | 1 | 17573 | UGSB 9303 |  | KX585155 |
|  |  |  | DC13/8 | 1 | 17574 | UGSB 9304 |  | KX585156 |
| DC14 | L | 35.37106 / 98.49651 | DC14/1 | 9 | 17399 | UGSB 8997 |  | KX585157 |
|  |  |  | DC14/2 | 9 | 17400 | UGSB 8998 |  | KX585158 |
|  |  |  | DC14/3 | 1 | 17401 | UGSB 8999 |  | KX585159 |
|  |  |  | DC14/4 | 9 | 17402 | UGSB 9000 |  | KX585160 |
|  |  |  | DC14/5 | 9 | 17619 | UGSB 9349 |  | KX585161 |
|  |  |  | DC14/6 | 1 | 17620 | UGSB 9350 |  | KX585162 |
|  |  |  | DC14/7 | 9 | 17621 | UGSB 9351 |  | KX585163 |
|  |  |  | DC14/8 | 9 | 17622 | UGSB 9352 |  | KX585164 |
| DC15 | L | 35.38115 / 98.47440 | DC15/1 | 1 | 17591 | UGSB 9321 |  | KX585165 |
|  |  |  | DC15/2 | 1 | 17592 | UGSB 9322 |  | KX585166 |
|  |  |  | DC15/3 | 1 | 17593 | UGSB 9323 |  | KX585167 |
|  |  |  | DC15/4 | 1 | 17594 | UGSB 9324 |  | KX585168 |
|  |  |  | DC15/5 | 1 | 17595 | UGSB 9325 |  | KX585169 |
|  |  |  | DC15/6 | 1 | 17596 | UGSB 9326 |  | KX585170 |
|  |  |  | DC15/7 | 1 | 17597 | UGSB 9327 |  | KX585171 |
|  |  |  | DC15/8 | 1 | 17598 | UGSB 9328 |  | KX585172 |
| DC16 | L | 35.38277 / 98.45560 | DC16/1 | 9 | 17403 | UGSB 9001 |  | KX585173 |
|  |  |  | DC16/2 | 9 | 17404 | UGSB 9002 |  | KX585174 |
|  |  |  | DC16/3 | 9 | 17405 | UGSB 9003 |  | KX585175 |
|  |  |  | DC16/4 | 1 | 17406 | UGSB 9004 |  | KX585176 |
| DC17 | DS | 35.37305 / 98.49303 | DC17/1 | 1 | 17575 | UGSB 9305 |  | KX585177 |
|  |  |  | DC17/2 | 1 | 17576 | UGSB 9306 |  | KX585178 |
|  |  |  | DC17/3 | 1 | 17577 | UGSB 9307 |  | KX585179 |
|  |  |  | DC17/4 | 1 | 17578 | UGSB 9308 |  | KX585180 |
|  |  |  | DC17/5 | 1 | 17579 | UGSB 9309 |  | KX585181 |
|  |  |  | DC17/6 | 1 | 17580 | UGSB 9310 |  | KX585182 |
| DC18 | DS | 35.28288 / 98.71648 | DC18/1 | 9 | 17407 | UGSB 9005 | √ | KX585183 |
|  |  |  | DC18/2 | 9 | 17409 | UGSB 9007 | √ | KX585185 |
|  |  |  | DC18/3 | 9 | 17410 | UGSB 9008 | √ | KX585186 |
|  |  |  | DC18/4 | 9 | 17623 | UGSB 9353 | √ | KX585187 |
|  |  |  | DC18/5 | 9 | 17625 | UGSB 9355 | √ | KX585188 |
|  |  |  | DC18/6 | 9 | 17626 | UGSB 9356 | √ | - |
|  |  |  | DC18/7 | 9 | 18786 | UGSB 11070 | √ | KX585189 |
|  |  |  | DC18/8 | 9 | 18787 | UGSB 11071 | √ | KX585190 |
|  |  |  | DC18/9 | 9 | 18790 | UGSB 11074 | √ | KX585191 |
|  |  |  | DC18/10 | 9 | 18791 | UGSB 11075 | √ | KX585184 |
| DC20 | DS | 35.19441 / 98.61356 | DC20/1 | 13 | 17411 | UGSB 9009 |  | KX585192 |
|  |  |  | DC20/2 | 13 | 17412 | UGSB 9010 |  | KX585193 |
|  |  |  | DC20/3 | 9 | 17413 | UGSB 9011 |  | KX585194 |
|  |  |  | DC20/4 | 9 | 17628 | UGSB 9358 |  | KX585195 |
|  |  |  | DC20/5 | 13 | 17629 | UGSB 9359 |  | KX585196 |
| DC21 | DS | 35.19160 / 98.64289 | DC21/1 | 9 | 17583 | UGSB 9313 | √ | KX585197 |
|  |  |  | DC21/2 | 9 | 17584 | UGSB 9314 | √ | KX585199 |
|  |  |  | DC21/3 | 9 | 17586 | UGSB 9316 | √ | KX585200 |
|  |  |  | DC21/4 | 9 | 17587 | UGSB 9317 | √ | KX585201 |
|  |  |  | DC21/5 | 9 | 17588 | UGSB 9318 | √ | KX585202 |
|  |  |  | DC21/6 | 9 | 17589 | UGSB 9319 | √ | - |
|  |  |  | DC21/7 | 9 | 17590 | UGSB 9320 | √ | - |
|  |  |  | DC21/8 | 9 | 18764 | UGSB 11048 | √ | KX585203 |
|  |  |  | DC21/9 | 9 | 18766 | UGSB11050 | √ | KX585204 |
|  |  |  | DC21/10 | 9 | 18768 | UGSB 11052 | √ | KX585198 |
| CN12 | L | 35.37019 / 98.49866 | CN12/1* | 9 | 10068 | UGSB 7368 |  | JN794401 |
|  |  |  | CN12/2* | 9 | 10069 | UGSB 7369 |  | JN794402 |
|  |  |  | CN12/3* | 9 | 10070 | UGSB 7370 |  | JN794403 |
|  |  |  | CN12/4* | 9 | 10935 | UGSB 7371 |  | JN794404 |
|  |  |  | CN12/5* | 9 | 10936 | UGSB 7372 |  | JN794405 |
|  |  |  | CN12/6* | 9 | 10937 | UGSB 7373 |  | JN794406 |
| CN13 | L | 35.38323 / 98.46711 | CN13/1* | 9 | 10938 | UGSB 7374 |  | JN794407 |
|  |  |  | CN13/2* | 9 | 10939 | UGSB 7375 |  | JN794408 |
|  |  |  | CN13/3* | 9 | 10940 | UGSB 7376 |  | JN794409 |
|  |  |  | CN13/4* | 9 | 10941 | UGSB 7377 |  | JN794410 |
|  |  |  | CN13/5* | 9 | 10942 | UGSB 7378 |  | JN794411 |
|  |  |  | CN13/6* | 9 | 10943 | UGSB 7379 |  | JN794412 |
| CN14 | L | 35.25400 / 98.50352 | CN14/1* | 9 | 10944 | UGSB 7380 |  | JN794413 |
| CN15 | DS | 35.19938 / 98.60763 | CN15/1* | 9 | 10071 | UGSB 7381 |  | JN794414 |
|  |  |  | CN15/2* | 9 | 10072 | UGSB 7382 |  | JN794415 |
|  |  |  | CN15/3* | 9 | 10073 | UGSB 7383 |  | JN794416 |
|  |  |  | CN15/4* | 9 | 10945 | UGSB 7384 |  | JN794417 |
|  |  |  | CN15/5* | 9 | 10946 | UGSB 7385 |  | JN794418 |
|  |  |  | CN15/6* | 9 | 10947 | UGSB 7386 |  | JN794419 |
|  |  |  | CN15/7* | 9 | 10948 | UGSB 7387 |  | JN794420 |
