# Supplementary material for: A Complex System of Glacial Sub-Refugia Drives Endemic Freshwater Biodiversity on the Tibetan Plateau
Source: PLoS One. 2016 Aug 8;11(8):e0160286. doi: 10.1371/journal.pone.0160286 (PMC4976922; doi:10.1371/journal.pone.0160286)
Supplement: S2 Table — (DOCX) [file pone.0160286.s003.docx]

**Table S2.** **Information on adapters and PCR primer sequences used for AFLP genotyping *Radix* specimens.**

| **Primer name** | **Primer sequence** |
| --- | --- |
| Adapter |  |
| *EcoR*I Forward | 5‘-CTC GTA GAC TGC GTA CC-3‘ |
| *EcoR*I Reverse | 5‘-AAT TGG TAC GCA GTC TAC-3‘ |
| *Mse*I Forward | 5‘-GAC GAT GAG TCC TGA G-3‘ |
| *Mse*I Reverse | 5‘-TAC TCA GGA CTC AT-3‘ |
| Pre-selective primers |  |
| *EcoR*I-A | 5‘-GAC TGC GTA CCA ATT CA-3‘ |
| *Mse*I-C | 5‘-GAT GAG TCC TGA GTA AC-3‘ |
| *Mse*I-AC | 5‘-GAT GAG TCC TGA GTA AAC-3‘ |
| Selective primers |  |
| IRD-800 *EcoR*I-AAC | 5‘-GAC TGC GTA CCA ATT CAA C-3‘ |
| IRD-700 *EcoR*I-AGC | 5‘-GAC TGC GTA CCA ATT CAG C-3‘ |
| *Mse*I-CAC | 5‘-GAT GAG TCC TGA GTA ACA C-3‘ |
| *Mse*I-CAT | 5‘-GAT GAG TCC TGA GTA ACA T-3‘ |
| *Mse*I-CGT | 5‘-GAT GAG TCC TGA GTA ACG T-3‘ |
| *Mse*I-ATC | 5‘-GAT GAG TCC TGA GTA AAT C-3‘ |
| *Mse*I-ACT | 5‘-GAT GAG TCC TGA GTA AAC T-3‘ |
| *Mse*I-ACTA | 5‘-GAT GAG TCC TGA GTA AAC TA-3‘ |
| *Mse*I-ACAT | 5‘-GAT GAG TCC TGA GTA AAC AT-3‘ |
